# High-throughput measurement of fibroblast rhythms reveals genetic heritability of circadian phenotypes in diversity outbred mice and their founder strains

**DOI:** 10.1038/s41598-021-82069-8

**Published:** 2021-01-28

**Authors:** Sam-Moon Kim, Chelsea A. Vadnie, Vivek M. Philip, Leona H. Gagnon, Kodavali V. Chowdari, Elissa J. Chesler, Colleen A. McClung, Ryan W. Logan

**Affiliations:** 1 Translational Neuroscience Program, Department of Psychiatry, University of Pittsburgh School of Medicine, 450 Technology Drive, Pittsburgh, PA 15219 USA; 2 Center for Systems Neurogenetics of Addiction, The Jackson Laboratory, 600 Main Street, Bar Harbor, 04609 ME USA; 3 Department of Pharmacology and Experimental Therapeutics, Boston University School of Medicine, 700 Albany Street, Boston, 02118 MA USA

**Keywords:** Genetics, Molecular biology, Neuroscience

## Abstract

Circadian variability is driven by genetics and Diversity Outbred (DO) mice is a powerful tool for examining the genetics of complex traits because their high genetic and phenotypic diversity compared to conventional mouse crosses. The DO population combines the genetic diversity of eight founder strains including five common inbred and three wild-derived strains. In DO mice and their founders, we established a high-throughput system to measure cellular rhythms using in vitro preparations of skin fibroblasts. Among the founders, we observed strong heritability for rhythm period, robustness, phase and amplitude. We also found significant sex and strain differences for these rhythms. Extreme differences in period for molecular and behavioral rhythms were found between the inbred A/J strain and the wild-derived CAST/EiJ strain, where A/J had the longest period and CAST/EiJ had the shortest. In addition, we measured cellular rhythms in 329 DO mice, which displayed far greater phenotypic variability than the founders—80% of founders compared to only 25% of DO mice had periods of ~ 24 h. Collectively, our findings demonstrate that genetic diversity contributes to phenotypic variability in circadian rhythms, and high-throughput characterization of fibroblast rhythms in DO mice is a tractable system for examining the genetics of circadian traits.

## Introduction

Circadian rhythms are found in most biological processes from biochemistry to behavior. In cells, rhythms are controlled by a series of transcriptional—translational feedback loops that cycle near 24 h, forming the molecular clock. The core genes and their proteins that comprise the molecular clock are expressed in nearly every cell in the body. Several transcription factors including CLOCK and BMAL1 drive the positive limb of the molecular clock^1^ and control the transcription of hundreds, if not thousands, of genes across different tissues and cell-types^2^. Depending on the tissue or cell-type, additional genes may modulate the ‘timing’ of the clock.

Efforts to identify new genes or variants that modulate the amplitude, phase, or period of the clock have heavily relied on mice to leverage their genetic diversity and availability of genetics tools^3,4^. While these efforts have been valuable, many of these studies have used common inbred mouse strains or conventional inbred crosses that are constrained by their narrow genetic and phenotypic range. Limited genetic and phenotypic diversity is a disadvantage for discovery of genetic drivers of complex traits^5^. The Diversity Outbred (DO) mice and companion inbred Collaborative Cross (CC) strains provide expansive genetic diversity and phenotypic heterogeneity, which has the potential to be tractable, powerful tools for investigating the genetics of circadian regulation^6^.

With the intention to recapitulate the genetic complexity in humans, the DO mice and CC strains were derived from an eight-way cross of five common inbred strains (A/J, C57BL/6J, 129S1/SvlmJ, NOD/ShiLtJ and NZO/HILtJ) and three wild-derived strains (CAST/EiJ, PWK/PhJ and WSB/EiJ). To begin to investigate whether the DO and CC mouse populations will be valuable for understanding the genetics of circadian rhythms, we established a high-throughput experimental system for measuring cellular rhythms in skin fibroblasts from the eight founder strains and a large cohort of DO mice. Uncovering novel genetic mechanisms of circadian rhythms may be translationally relevant for further understanding human circadian biology, as many circadian phenotypes (*e.g.,* period) are heritable with few insights into their genetic bases^6–9^.

For more than a decade, skin fibroblast preparations have been a highly efficient and reliable approach for measuring cellular and molecular rhythms in vitro^10,11^. Real-time measurement of circadian gene bioluminescent reporters can be measured over days. Remarkably, the molecular rhythms of skin fibroblasts are largely consistent with the circadian pacemaker of the suprachiasmatic nucleus (SCN)^12^, and also locomotor activity rhythms, when measured in the same mouse^13^. Skin fibroblasts have been successfully leveraged to conduct large-scale mutagenesis screens to identify genes with novel roles in circadian rhythms^14^. In addition, fibroblasts have been used to conduct screens of thousands of small molecular compounds that alter rhythms^15^. In humans, fibroblasts have been reliable for identifying molecular mechanisms of rhythm abnormalities related to psychiatric disorders^16–18^. Thus, measuring fibroblast rhythms is a robust approach for investigating the genetics of circadian regulation from mice to humans.

In the present study, we used skin fibroblasts from male and female mice of the eight founder strains of CC and DO mice, and a large cohort of DO mice, to investigate the impact of genetic variation on molecular rhythms. Across the founder strains, we observed strain-dependent variability in several circadian measures, which contributed to modest heritability estimates for circadian period, phase, and amplitude. In DO mice, we found pronounced variation of period, phase, and amplitude. Notably, the variation of several of these measures in DO mice expanded beyond the bounds of the founder strains. These findings support the utility of DO mice for high-precision genetics of circadian rhythms.

## Results

### Strain-dependent differences in molecular rhythm phenotypes

**Figure 1:**
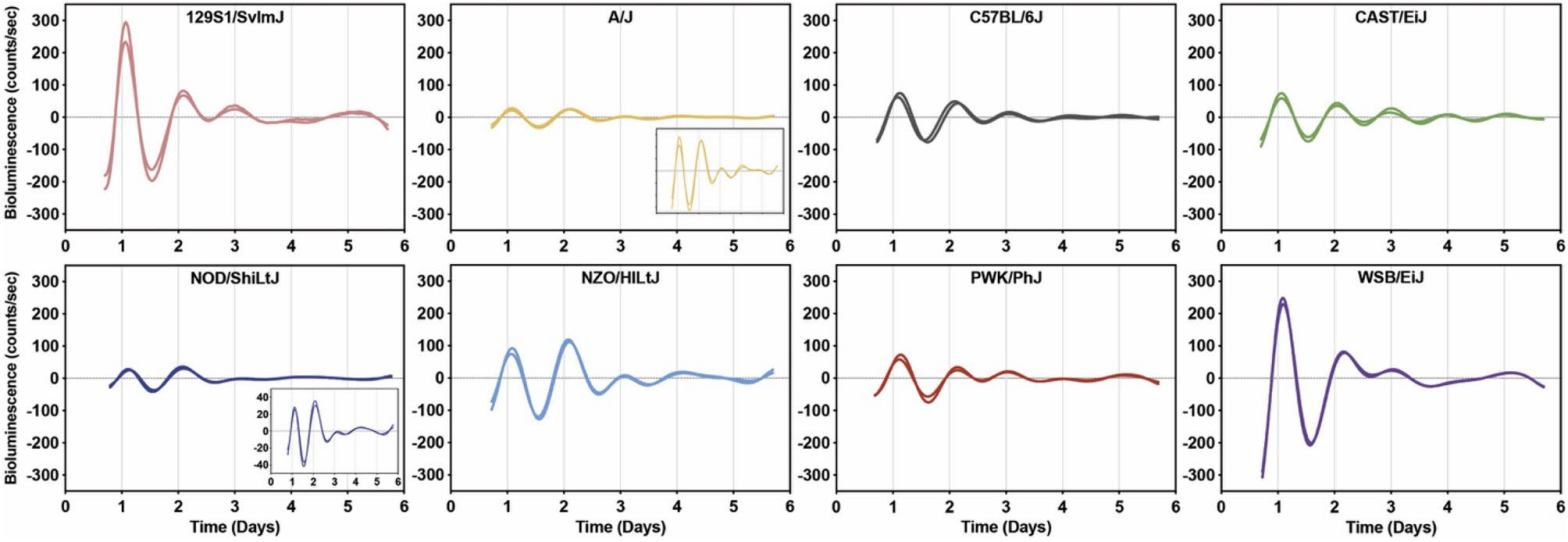
Representative bioluminescence recordings of ensemble *Bmal1-dLuc* rhythms in two individual fibroblast cultures derived from eight founder strains plotted by baseline-subtracted data.

To investigate the association between genetic diversity and circadian rhythms, we measured molecular rhythms in primary skin fibroblast cultures derived from mice of the eight founder strains of the DO mouse population. Fibroblast cultures were synchronized using forskolin, an activator of cAMP signaling and Ca^2+^/cAMP responsive element binding protein^19^. After forskolin-induced synchronization, fibroblast cultures displayed strong rhythmic oscillations of *Bmal1-dLuc* bioluminescence for more than 3–4 cycles (Fig. 1, Supplementary Fig. 1). The baseline-subtracted bioluminescence traces were relatively consistent between fibroblast cultures derived from different sampling cohorts within the same strain of mice (Fig. 1). We compared *Bmal1-dLuc* rhythms obtained from each mouse strain to the most common inbred strain used for biomedical research, C57BL/6J mice. We found significant differences for sex, strain, or sex by strain effects for fibroblast rhythm period, amplitude, phase, and goodness of fit (“robustness”).

**Figure 2:**
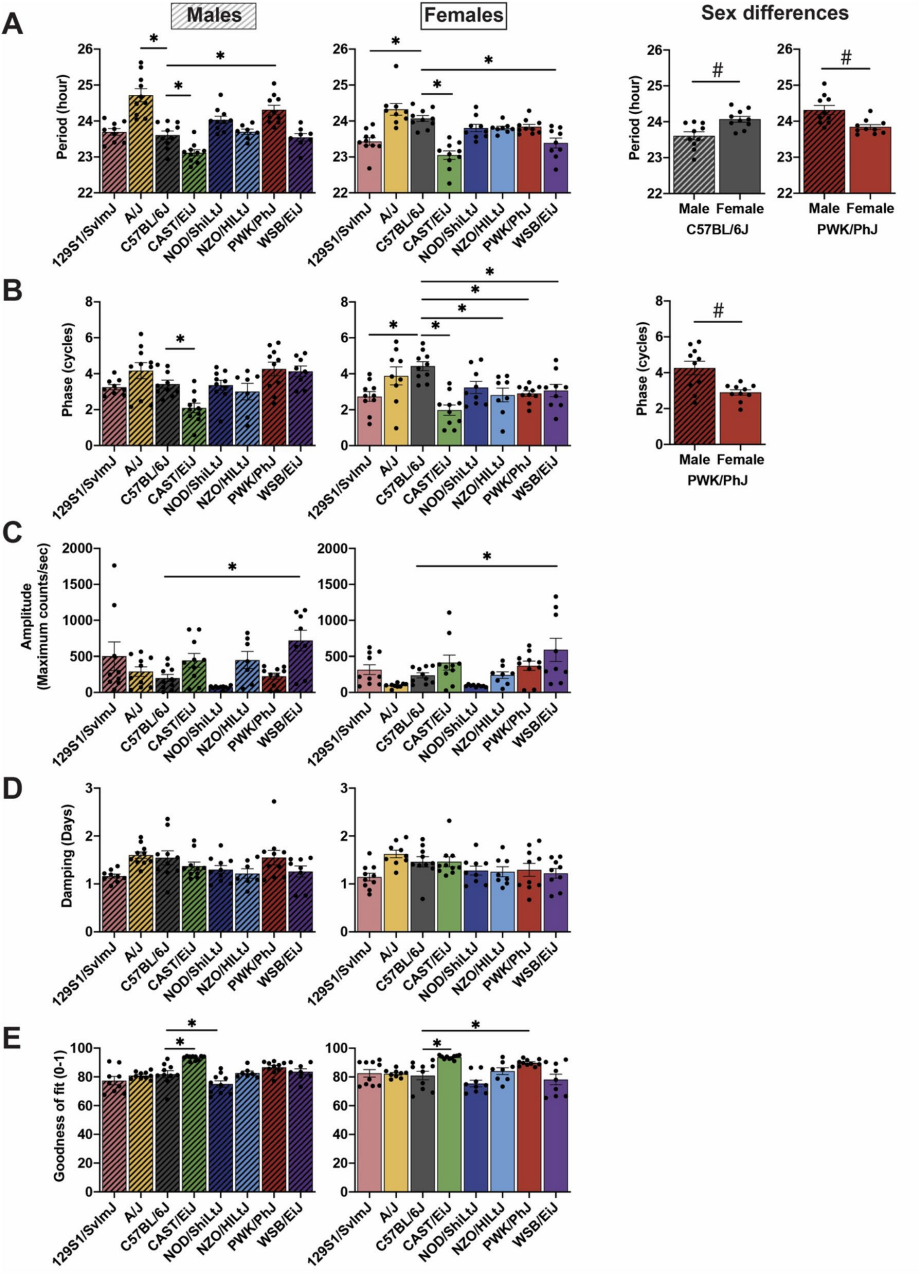
Strain and sex differences of ensemble *Bmal1-dLuc* rhythms in primary fibroblast cultures. Quantified data for strain and sex differences in **(A)** period, **(B)** phase, **(C)** amplitude, **(D)** damping rate and **(E)** goodness of fit of *Bmal1-dLuc* rhythms. Bar graphs depict mean ± SEM. Bar graphs with fill pattern indicate the data from males. Asterisks indicate that circadian parameters are significantly (P < 0.05) different compared to C57BL/6J within the same sex. Hashtags indicate significant (P < 0.05) sex differences in period and phase of *Bmal1-dLuc* rhythms.

In males, we found a significant strain by sex interaction (F_1,133_ = 3.578, P = 0.0015) for *Bmal1-dLuc* rhythm period in the founder strains. The fibroblast rhythm periods were significantly longer in A/J (24.72 ± 0.18 h, P < 0.0001) and PWK/PhJ (24.31 ± 0.13 h, P = 0.0003) mice and shorter in CAST/EiJ (23.12 ± 0.09 h, P = 0.0203) mice compared to C57BL/6J (23.61 ± 0.11 h) mice (Fig. 2A). In females, the periods were significantly shorter in 129S1/SvlmJ (23.43 ± 0.11 h P = 0.0004), CAST/EiJ (23.05 ± 0.12 h P < 0.0001) and WSB/EiJ (23.39 ± 0.14 h, P = 0.0002) mice compared to C57BL/6J (24.07 ± 0.08 h) mice (Fig. 2A). We also observed a significant sex difference for the periods of fibroblast rhythms in C57BL/6J and PWK/PhJ mice. In C57BL/6J mice, males displayed significantly shorter periods relative to females (P = 0.0279), while male PWK/PhJ mice had longer periods relative to females by ~ 30 min (Fig. 2A; P = 0.0230).

In addition, we observed strain by sex interactions (F_7,133_ = 2.539, P = 0.0175) in the peak phase of fibroblast rhythms. For each strain, peak phase was compared to C57BL/6J mice to determine whether the rhythms were advanced or delayed. In males, CAST/EiJ (2.09 ± 0.26 cycles) mice had a significantly advanced phase of rhythms relative to C57BL/6J (3.42 ± 0.22 cycles) mice (Fig. 2B). Similarly, female CAST/EiJ (1.98 ± 0.29 cycles) mice had an advanced phase than C57BL/6J (4.43 ± 0.25 cycles) mice (Fig. 2B). Female mice from other strains also had a significantly advanced phase compared to C57BL/6J mice, including 129S1/SvlmJ (2.74 ± 0.27 cycles), NZO/HiLtJ (2.82 ± 0.39 cycles), PWK/PhJ (2.90 ± 0.15 cycles), and WSB/EiJ (3.07 ± 0.34 cycles) mice (Fig. 2B). No strains displayed significantly delayed phases compared to C57BL/6J mice (Fig. 2B). Additionally, a sex difference was observed in PWK/PhJ (P = 0.0190) mice displaying a significant phase advance of *Bmal1-dLuc* rhythms in females compared to males by 1.36 cycles (Fig. 2B), consistent with a marked shorter period of molecular rhythms in females relative to males in this wild-derived strain.

We also observed significant strain differences in amplitude and goodness of fit of fibroblast rhythms. Goodness of fit is a measure derived from the overall fit of the waveform to the bioluminescence rhythms, where higher values represent more rhythm “robustness”. In WSB/EiJ mice, amplitudes were ~ 3.6-fold and ~ 2.5-fold higher compared to C57BL/6J mice in males and females, respectively (Fig. 2C). In males, CAST/EiJ (93.27 ± 0.49) mice had significantly higher values, while NOD/ShiLtJ (75.03 ± 2.16) mice had significantly lower values  in goodness of fit compared to C57BL/6J (81.86 ± 2.43) mice (Fig. 2E). In females, CAST/EiJ (93.69 ± 0.44) and PWK/PhJ (89.81 ± 0.69) mice had significantly higher goodness of fit than C57BL/6J (81.08 ± 2.96) mice (Fig. 2E). In addition, there were no significant effects of sex, or strain by sex interactions for amplitude or goodness of fit.

Moreover, we measured damping rate to assess rhythm “persistence”. Damping rate measures the time for the rhythm to decay to half its original amplitude. We did not observe any strain and sex effects for the damping rate of fibroblast rhythms in the founder strains indicating all fibroblast cultures displayed similar persistence in their rhythms over 3–4 days (Fig. 1 and 2D).

### Genetic heritability of fibroblast rhythms

Many of the fibroblast rhythm measures were highly variable among the founder strains. To determine whether this rhythm variability is caused by genetic differences among the founder strains, we calculated heritability (*h*^*2*^) as the percentage of variability attributed to strain differences across the founders. To understand the genetic effects, similar culture conditions were used across each of the samples and experiments, including cell number and transfection efficiencies. Our analyses revealed that fibroblast rhythm period, phase, amplitude and goodness of fit were heritable (Table 1). The highest heritability was rhythm period (35% strain-dependent variability), followed by goodness of fit (33%), amplitude (26%), and phase (26%). Damping rate was the lowest at 13% (Table 1). While these heritability estimates were modest, nevertheless, our findings demonstrate that these founder strains capture genetic diversity that contributes to phenotypic variability in molecular rhythm phenotypes.

**Table 1:** Heritability estimates of circadian parameters of *Bmal1-dLuc* rhythms in cultured fibroblasts derived from the founder strains.

Circadian parameters	% Heritability (h^2^)
Period	35%
Phase	26%
Amplitude	26%
Damping rate	13%
Goodness of fit	33%

### Expansion of rhythm phenotypic variability in DO mice relative to the founder strains

Based on previous studies^5^, we expected higher variability of fibroblast rhythms in DO mice that would extend beyond the range observed in the founder strains. To investigate this, we measured fibroblast rhythms in a large sample of male and female DO mice (n = 329). We then compared the distributions of fibroblast rhythms parameters between the DO mice and the founder strains.

**Figure 3:**
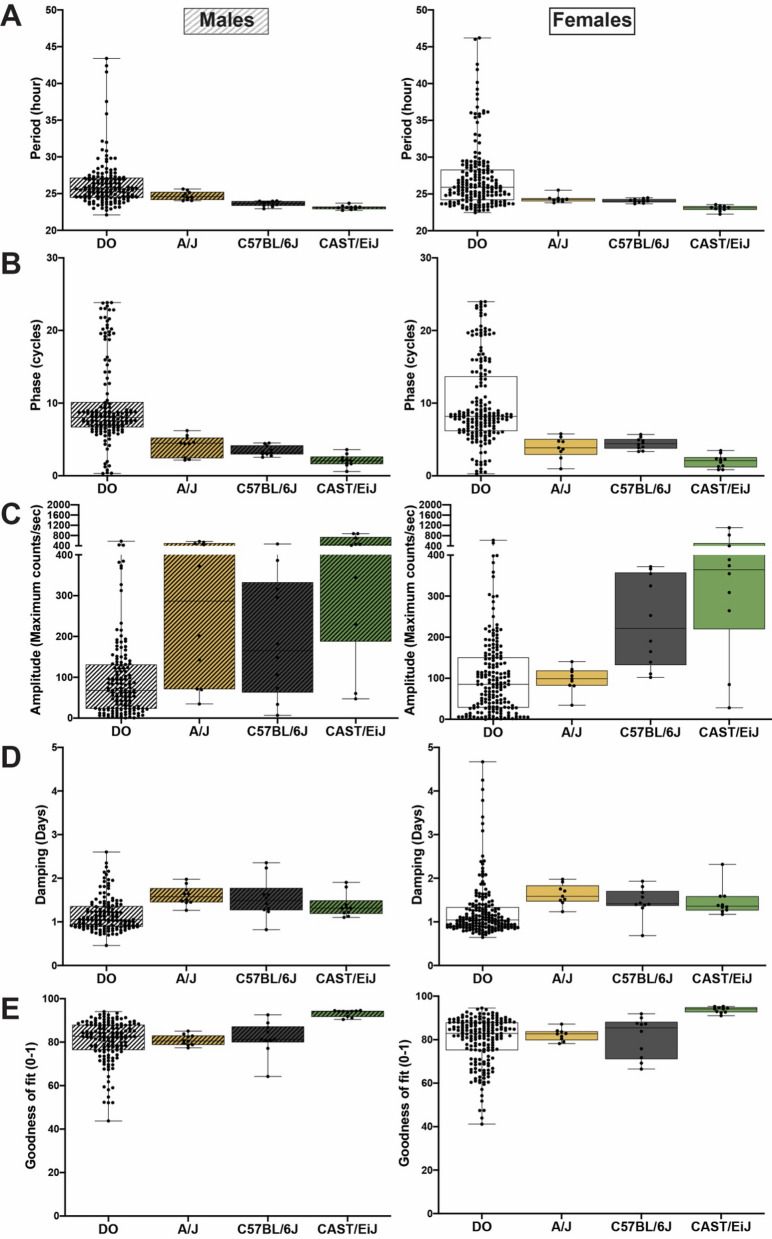
Distribution of circadian parameters in DO mice and two extreme founder strains, A/J and CAST/EiJ, along with C57BL/6J strain. **(A)** Period, **(B) **phase, **(C)** amplitude, **(D)** damping rate and **(E)** goodness of fit of ensemble *Bmal1-dLuc* rhythms in primary fibroblast cultures. Lines in bars indicate median and the whiskers indicate Min to Max.

In the founder strains, the range for period was similar between males (22.72 h-25.63 h) and females (22.26 h-25.53 h), with ~ 80% of mice displaying rhythms near 24 h (Fig. 3A, Table 2). A/J and CAST/EiJ represented either ends of the phenotypic distribution, where A/J had the longest period and CAST/EiJ had the shortest period (Figs. 2A and 3A). As expected, DO mice displayed an expanded range of variability in period of fibroblast rhythms. Only 24% of DO mice displayed rhythm periods near 24 h (Fig. 3A, Table 2). The period range for DO mice spanned more than 20 h in males (22.11 h-43.40 h) and females (22.47 h-46.2 h) (Fig. 3A, Table 2).

**Table 2:** Summary of circadian parameters of *Bmal1-dLuc* rhythms in cultured fibroblasts derived from DO mice and their founder strains. Asterisks indicate that circadian parameters are significantly (P < 0.05) different compared to C57BL/6J within the same sex. Hashtags indicate significant (P < 0.05) sex differences in period and phase of *Bmal1-dLuc* rhythms.

Strain	Sex	N	Period		Phase		Amplitude		Damping rate		Goodness of fit	
			Mean ± SEM	Min–max	Mean ± SEM	Min–max	Mean ± SEM	Min–max	Mean ± SEM	Min–max	Mean ± SEM	Min–max
129S1/SvlmJ	Male	9	23.70 ± 0.09	23.29–24.08	3.25 ± 0.15	2.75–4.07	502.15 ± 196.37	39.66–1762.68	1.16 ± 0.04	0.96–1.37	77.40 ± 2.91	67.56–90.79
	Female	10	23.43 ± 0.11*	22.68–23.91	2.74 ± 0.27*	1.20–3.99	316.27 ± 67.27	83.93–623.51	1.14 ± 0.08	0.78–1.64	82.51 ± 2.61	73.29–91.96
A/J	Male	10	24.72 ± 0.18*	24.04–25.63	4.17 ± 0.45	2.16–6.21	290.23 ± 65.73	34.49–563.32	1.60 ± 0.07	1.26–1.98	80.97 ± 0.79	77.39–85.10
	Female	9	24.32 ± 0.16	23.80–25.53	3.88 ± 0.50	0.97–5.79	97.18 ± 10.06	34.49–140.38	1.62 ± 0.08	1.23–1.98	82.15 ± 0.92	78.21–87.17
C57BL/6J	Male	10	23.61 ± 0.11#	22.94–24.00	3.42 ± 0.22	2.55–4.50	201.51 ± 49.72	6.91–469.26	1.55 ± 0.15	0.82–2.36	81.86 ± 2.43	64.21–92.55
	Female	10	24.07 ± 0.08	23.68–24.48	4.43 ± 0.25	3.35–5.70	237.74 ± 34.61	102.04–371.70	0.46 ± 0.11	0.69–1.93	81.08 ± 2.96	66.49–91.91
CAST/EiJ	Male	10	23.12 ± 0.09*	22.72–23.70	2.09 ± 0.26*	0.59–3.59	444.83 ± 93.93	47.29–872.76	1.37 ± 0.09	1.10–1.91	93.27 ± 0.49*	90.45–94.58
	Female	10	23.05 ± 0.12*	22.26–23.55	1.98 ± 0.29*	0.85–3.47	413.44 ± 102.48	28.16–1107.32	1.46 ± 0.10	1.17–2.32	93.69 ± 0.44*	91.01–95.28
NOD/ShiLtJ	Male	10	24.03 ± 0.10	23.64–24.73	3.37 ± 0.27	1.34–4.36	70.39 ± 6.96	20.22–101.17	1.30 ± 0.08	0.97–1.80	75.03 ± 2.16*	65.96–85.99
	Female	9	23.80 ± 0.10	23.42–24.39	3.24 ± 0.34	2.18–4.79	91.90 ± 4.90	70.89–116.18	1.28 ± 0.09	0.95–1.82	75.44 ± 2.30	68.38–86.62
NZO/HILtJ	Male	7	23.70 ± 0.07	23.38–23.96	3.00 ± 0.46	1.10–4.16	449.63 ± 116.57	50.16–824.66	1.22 ± 0.10	0.84–1.57	82.75 ± 1.32	79.60–89.80
	Female	8	23.80 ± 0.05	23.59–24.10	2.82 ± 0.38*	0.79–4.79	242.10 ± 45.75	49.37–443.15	1.25 ± 0.10	0.92–1.77	84.02 ± 2.39	73.30–93.86
PWK/PhJ	Male	10	24.31 ± 0.13*#	23.82–25.04	4.26 ± 0.38#	2.34–5.72	226.07 ± 42.01	16.12–365.05	1.55 ± 0.15	1.08–2.72	86.68 ± 1.33	77.13–90.94
	Female	10	23.84 ± 0.06	23.63–24.28	2.90 ± 0.15*	1.94–3.52	371.52 ± 64.06	30.89–649.57	1.29 ± 0.13	0.67–1.90	89.81 ± 0.69*	86.83–93.49
WSB/EiJ	Male	8	23.54 ± 0.10	22.97–23.96	4.13 ± 0.30	3.02–5.15	718.73 ± 144.19*	116.78–1140.48	1.26 ± 0.12	0.76–1.54	83.56 ± 2.03	73.11–90.29
	Female	9	23.39 ± 0.14*	22.65–23.86	3.07 ± 0.34*	1.48–4.76	590.63 ± 159.36*	117.69–1331.38	1.22 ± 0.10	0.74–1.57	78.23 ± 3.67	65.38–92.02
Diversity outbred	Male	142	26.32 ± 0.28	22.11–43.40	9.89 ± 0.49	0.32–23.84	94.62 ± 8.33	0.58–578.85	1.17 ± 0.03	0.46–2.60	80.82 ± 0.80	43.76–94.05
	Female	187	27.17 ± 0.32	22.47–46.20	9.94 ± 0.42	0.28–23.98	104.30 ± 7.21	0.15–622.96	1.25 ± 0.05	0.65–4.67	79.68 ± 0.78	41.15–94.55

Variability of rhythm phase was also limited in the founder strains compared to DO mice. Across the founders, the range for males was 0.59 to 6.21 cycles and from 0.79 to 5.79 cycles in females (Figs. 2B and 3B, Table 2). A/J mice displayed the most delayed rhythm, while CAST/EiJ mice had the most advanced rhythm, consistent with their differences in period length between two strains (Figs. 2B and 3B). Most of the DO mice displayed rhythm phase between 6 to 9 cycles in males (64%) and between 5 and 9 cycles in females (57%), although cycles varied from 0 to 24 cycles across all DO mice (Fig. 3B, Table 2).

In contrast to period and phase, amplitude variability in DO mice was comparatively less to the founder strains (Fig. 3C, Table 2). On average, amplitude was lower in DO mice than that in the founder strains (P < 0.0001). In addition, the damping rate and goodness of fit measures were similarly distributed in the founder strains and DO mice (Figs. 3D and 3E, Table 2). Overall, the DO mice displayed more phenotypic diversity in fibroblast rhythms than the founder strains, indicating an expansion of rhythm variability phenotypes because of their high genetic diversity.

### Correlations between fibroblast rhythm periods and phases in DO mice and their founder strains

The circadian period and phase are strongly correlated to each other—more delayed or advanced rhythm is associated with a longer or shorter period, respectively^20–23^. Consistent with this, A/J mice had the most delayed phase and also the longest period, while CAST/EiJ mice displayed the most advanced rhythm and the shortest period length among the founder strains (Figs. 2A and 2B). To investigate further, we correlated period and phase in DO mice and their eight founder strains. As expected, mice with a longer period displayed more delayed rhythms, and vice versa (Supplementary Fig. 2). This correlation was stronger in females (R^2^ = 0.3336, r = 0.5776, P < 0.0001 and n = 262) than males (R^2^ = 0.1360, r = 0.3688, P < 0.0001 and n = 216) in DO mice  and their founder strains (Supplementary Fig. 2).

### Similarity of molecular and behavioral rhythms in extreme founder strains

Cellular and molecular clocks modulate physiological and behavioral circadian rhythms^24^. Therefore, we investigated whether fibroblast rhythms were consistent with locomotor activity rhythms in the founder strains. Since the highest heritability estimate was circadian period, we investigated behavioral rhythm period in the two founder strains with the most extreme differences in molecular rhythm period, along with C57BL/6J strain as a reference. Among the founder strains, the inbred A/J (24.72 ± 0.18 h and 24.33 ± 0.16 h for males and females, respectively) and the wild-derived CAST/EiJ (23.12 ± 0.09 h and 23.05 ± 0.12 h for males and females, respectively) strains were on opposite ends of the period distribution in fibroblast rhythms (Fig. 2A). In comparison with C57BL/6J mice (23.61 ± 0.11 h and 24.07 ± 0.08 h for males and females, respectively), the fibroblast rhythm periods were significantly shorter in CAST/EiJ mice in both sexes, but longer only in A/J males (Fig. 2A).

**Figure 4:**
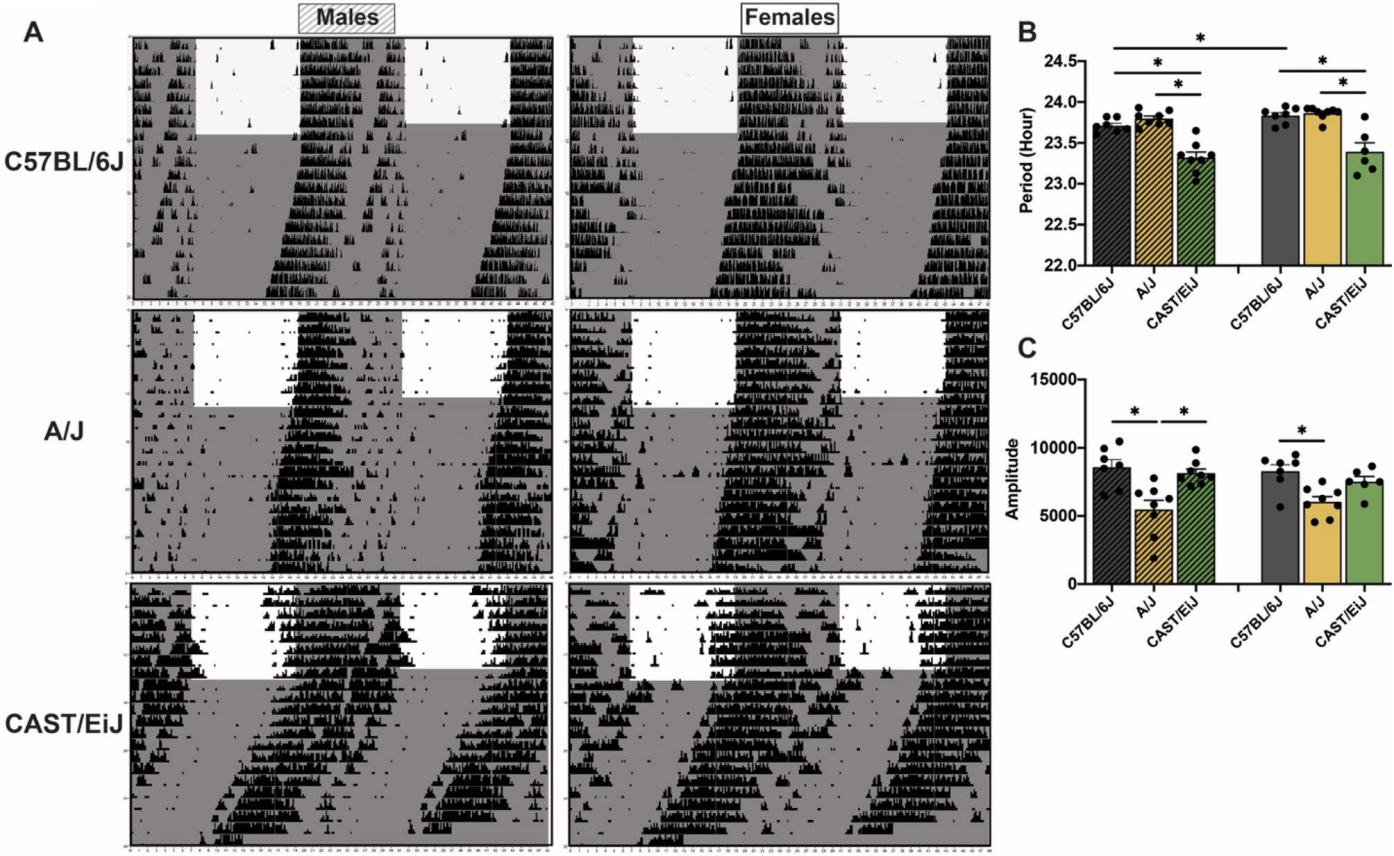
Circadian rhythms of wheel-running activity in two extreme founder strains along with C57BL/6J strain. **(A)** Representative actograms of wheel-running activity in C57BL/6J, A/J and CAST/EiJ mice that were maintained under 12:12 light–dark cycles for entrainment and then released to constant darkness for free-run. **(B)** The period and **(C)** amplitude of the activity rhythms during constant darkness were quantified by periodogram analysis. Shade indicates light-off. Bar graphs depict mean ± SEM. Bar graphs with fill pattern indicate the data from males. Asterisks indicate significant differences (p < 0.05).

A/J and C57BL/6J mice reliably entrained to standard 12:12 light–dark cycles with activity onsets beginning at the start of dark phase (Fig. 4A). In contrast, CAST/EiJ mice started their activity 2–3 h before the dark phase (Fig. 4A). CAST/EiJ (23.32 ± 0.07 h and 23.39 ± 0.11 h for males and females, respectively) mice exhibited a significantly shorter free-running period of activity rhythms compared to C57BL/6J (23.71 ± 0.03 h and 23.83 ± 0.04 h for males and females, respectively) and A/J (23.80 ± 0.03 h and 23.86 ± 0.03 h for males and females, respectively) mice during constant darkness (Figs. 4A and 4B). A/J displayed a trend of longer free-running period in males (P = 0.0754), but not in females, compared to C57BL/6J (Figs. 4A and 4B). We also observed a small, but significant sex difference for free-running period of activity rhythms in C57BL/6J, where males displayed significantly shorter periods relative to females (P = 0.0288) (Figs. 4A and 4B), consistent with those observed in their fibroblast rhythms (Fig. 2A). There were no significant sex effects in A/J and CAST/EiJ mice. In addition, we found a significant strain difference in the amplitude of wheel-running activity under constant darkness. We found a 156% and 137% difference in amplitude in C57BL/6J males (P = 0.0021) and females (P = 0.0029) relative to A/J mice respectively (Fig. 4C). CAST/EiJ showed 148% higher amplitude of activity rhythms than A/J (P = 0.0029) in males (Fig. 4C). Our findings demonstrate similar rhythm phenotypes in fibroblasts and behavior in mice we identified as the most extreme of the founder strains.

## Discussion

Fibroblasts are a well-established experimental system for measuring circadian rhythms. Fibroblasts display robust molecular rhythms that can be measured via the expression of canonical circadian genes such as *Bmal1*. Previous studies demonstrated that primary fibroblasts display stable and persistent molecular rhythms^25–27^ that are self-sustained and cell-autonomous^27,28^. Furthermore, fibroblast rhythms largely approximate other measures of circadian rhythms including locomotor activity^13^. Reproducible molecular rhythms across fibroblast cultures made these cells an attractive and tractable system for high-throughput rhythm phenotyping in the founder strains and a large cohort of DO mice. Here, we found molecular rhythms were maintained across 3–4 cycles in fibroblast cultures and rhythms were largely reproducible in male and female mice within each of the founder strains. Between strains, rhythm phenotypes varied significantly, reflected by heritability estimates from 13% to 35% for damping rate and period, respectively. We also found that fibroblast rhythms were highly variable between individual DO mice, with many rhythm phenotypes expanding beyond the overall range of the founders. Phenotypic expansion is likely due to allelic heterozygosity of the DO mice^5,29^, with novel variants and allele mixtures driving the higher variability of rhythm phenotypes compared to inbred mouse strains. These findings suggest the DO mouse population and their founder strains are amenable to high precision genetic analysis of fibroblast rhythm phenotypes.

Genetic diversity is the primary driver for differences in molecular rhythms, which ultimately regulate physiological and behavioral rhythms. Previous studies have shown that genetic variants or mutations in canonical circadian genes lead to marked changes in circadian rhythm phenotypes across many different organisms^3,4,30^. Single base mutations in *Per2*^31^ or *Clock*^32^ genes altered period of behavioral rhythms in mice^31^. Our discovery that fibroblast rhythms are highly variable among founder strains and also individual DO mice, suggests that known or yet to be discovered variants could contribute to these strain-dependent differences. To begin to understand these possibilities, future studies will examine whether the phenotype variability could be attributed to variants in canonical circadian genes. Variants in factors known to impact the timing of the molecular clock could also explain these differences^1^.

Consistent with data from humans^33^, we demonstrated that circadian rhythm phenotypes were heritable traits in male and female mice from the founder strains of the DO mice. The heritability estimates indicate that the period, phase, amplitude, and goodness of fit differences in molecular rhythms across strains are largely due to genotypic variation among the founder strains. Interestingly, we found consistent rhythm periods measured in fibroblasts or using running wheels in two of the mouse strains with the most extreme circadian phenotypes among the founders, along with C57BL/6J strain. Similar to the period in fibroblast cultures, the periods of behavioral rhythms in CAST/EiJ mice were shorter than those in A/J and C57BL/6J mice. Our observation is entirely consistent with other studies that have reported shorter rhythm periods in male CAST/EiJ compared to C57BL/6J mice^34^. Shorter periods in CAST/EiJ mice were further supported by a more recent study which demonstrated strain-dependent differences in circadian wheel-running behavior^6^. Notably, the overall pattern of differences in circadian period among the founder strains were consistent with our findings, with the exception of A/J mice^6^. Others have also reported shorter periods in A/J mice relative to C57BL/6J mice^35,36^. This discrepancy between our observation that A/J mice have comparatively longer periods and these previous reports could be due to experimental conditions, such as illumination intensity during entrained 12:12 LD cycles^37^. Nevertheless, our data support longer periods in A/J mice, as both molecular and behavioral rhythms were significantly longer than C57BL/6J mice. The strain differences in period were more pronounced in fibroblasts than wheel-running behavior in A/J, CAST/EiJ and C57BL/6J mice, a relationship previously reported in circadian gene mutant mice^13,38^. In addition, we found similar sex effects on the rhythm periods in fibroblasts and wheel-running activity. The periods of behavioral rhythms in C57BL/6J females were longer than those in males as we observed in the fibroblast rhythm periods.

Both male and female CAST/EiJ mice exhibited an activity onset that occurred ~ 2–3 h prior to when the lights turned off. This suggests that CAST/EiJ have altered entrainment to environmental LD cycles, consistent with an attenuated response to the masking effects of light in this strain^34^. CAST/EiJ mice are one of the three wild-derived founder strains. Investigating whether attenuated light responsiveness is also found in other wild-derived strains could suggest shared genetic substrates of light entrainment pathways in the brain. Future studies will examine the impact of light-induced phase shifts and other changes to the LD cycle on the founder strains and DO mice.

Surprisingly, we discovered consistent sex differences in fibroblast rhythm phenotypes. Our fibroblast cultures were derived from skin biopsies then cultured for several weeks prior to bioluminescence recordings. Thus, we did not expect to see marked sex differences in fibroblast rhythms, as these cells are completely removed from any hormonal milieu. An intriguing possibility is that there are sex-specific genetic (i.e., XX vs. XY) or epigenetic mechanisms in the regulation of fibroblast rhythms. Potential serum derived hormones from culture medium seems less likely to have an impact since male and female fibroblasts were cultured using similar conditions. Ongoing and future studies will further examine the interaction between sex and strain on fibroblast rhythms.

In both male and female mice, we observed a consistent relationship between rhythm amplitude and shorter periods. The only exception was male NZO/HILtJ mice. While there are few studies directly examining the relationships between period and amplitude, there is evidence that a longer period is usually accompanied by lower amplitude rhythms^39^. Recently, we reported differences between CC strains, which are also derived from the eight founders, where one of the lines displayed a longer period and lower amplitude in both molecular and behavioral rhythms compared to another line^40^. This suggests that circadian amplitude and period may be modulated by common genetic substrates in certain mouse strains and CC lines. Another possibility for strain effects on amplitude observed in our study are differences in cell to cell communication that synchronizes rhythmic activity in the fibroblast cultures. Cell number and density in fibroblast cultures has been shown to influence rhythm amplitudes in vitro^41^. Although this is a potential factor in our studies, we controlled cell number and density by plating and transfecting equal cell numbers across each fibroblast culture for every strain and sex. Moreover, our recording conditions were devoid of serum and growth factors, mitigating the effect of strain-specific differences on cell proliferation rates during bioluminescence measurements.

Our present study indicates that different genetic backgrounds are associated with circadian variability in DO mice and their founder strains. Similar as other studies to understand genetic contribution to circadian variation in different model organisms including neurospora and fly^42–44^, we established high-throughput fibroblast screening of molecular rhythms as a tractable approach for beginning to identify new genetic mechanisms that contribute to natural variation in circadian rhythms in DO mice. With the support of these initial findings, future studies will use genetic mapping approaches to identify genetic loci that can be examined directly for their effects of circadian rhythm phenotypes. Since circadian disruption is considered a risk factor for many diseases ^45–47^, future studies will investigate the genetic contributions to both circadian rhythms and disease-related phenotypes in DO mice and their founder strains.

## Methods

### Animals

Male and female (8–12 weeks old) DO mice and each of their eight founder strains (A/J, C57BL/6J, 129S1/SvlmJ, NOD/ShiLtJ, NZO/HILtJ, CAST/EiJ, PWK/PhJ and WSB/EiJ) were used for fibroblast isolation and molecular rhythm analysis. DO mice and their founder strains were housed at The Jackson Laboratory under a 12:12 light–dark (LD) cycle (lights on at 6:00 am). To measure running-wheel activity rhythms, C57BL/6J (males: n = 7; females: n = 7), A/J (males: n = 8; females: n = 8) and CAST/EiJ (males: n = 8; females: n = 6–8) mice (8–12 weeks old) were delivered from The Jackson Laboratory and housed at University of Pittsburgh under a 12:12 LD cycle (lights on at 7:00 am), then allowed to habituate for at least one week prior to behavioral experiments. All mice were fed food and water ad libitum. All animal procedures used in this study abide by the ARRIVE (Animal Research: Reporting In Vivo Experiments) guidelines and were conducted in accordance with the National Institute of Health guidelines for the care and use of laboratory animals and approved by the Institutional Animal Care and Use Committee of the University of Pittsburgh.

### Fibroblast isolation

Primary fibroblast cultures were generated for all founder strains [A/J (males: n = 10; females: n = 9), C57BL/6J (males: n = 10; females: n = 10), 129S1/SvlmJ (males: n = 9; females: n = 10), NOD/ShiLtJ (males: n = 10; females: n = 9), NZO/HILtJ (males: n = 7; females: n = 8), CAST/EiJ (males: n = 10; females: n = 10), PWK/PhJ (males: n = 10; females: n = 10) and WSB/EiJ (males: n = 8; females: n = 9)] and DO (males: n = 142; females: n = 187) mice. To generate primary fibroblast cultures, an ear punch in 1 mm diameter was taken from each mouse (male and female, 8–12 weeks old) of DO mice and their founder strains at The Jackson Laboratory and then was shipped overnight in Dulbecco’s Modified Eagle’s Medium (DMEM) to the University of Pittsburgh. The tissues were digested in DMEM containing 2.5 mg/ml collagenase D (Gibco) and 1.25 mg/ml pronase (Millipore) for 90 min. The digested biopsy was plated in a 60 mm tissue culture dish and overlaid by sterilized 22 mm square cover glass in DMEM containing 10% fetal bovine serum (FBS, HyClone), 1% amphotericin B (Sigma) and 0.1% gentamycin (Gibco) and then maintained in a standard tissue culture incubator at 37 °C until *Bmal1-dLuc* reporter gene transduction.

### *Bmal1-dLuc* circadian rhythm measurement

To measure molecular rhythms from primary fibroblast cultures, 1 × 10^5^ cells were plated in a 35 mm tissue culture dish then transfected with 1 × 10^7^ units of lentiviral construct (VectorBuilder) expressing luciferase reporter fused to the *Bmal1* circadian gene (*Bmal1-dLuc,* Addgene). All cells were exposed to 15 µM forskolin (Sigma) shock for 2 h to facilitate synchronization across cultures^48^. Following synchronization, cells remained in DMEM media supplemented with 15 µM forskolin, 25 mM HEPES (Gibco), 292 µg/ml L-glutamine (HyClone), 100 units/ml penicillin (HyClone), 100 μg/ml streptomycin (HyClone) and 10 µM luciferin (Promega) during bioluminescence recording by an automated 32-channel luminometer (LumiCycle, Actimetrics) in a standard tissue culture incubator at 32 °C. Bioluminescence signals were recorded from 2–4 fibroblast cultures per each biological sample for 5–6 days every ~ 70 secs at intervals of 10 min.

Circadian parameters including period, amplitude, phase, damping rate and goodness of fit were determined from baseline-subtracted data using the damped sine fit and Levenberg–Marquardt algorithm by LumiCycle Analysis software ^25^. Briefly, the software estimated the peak-to-peak intervals as period length indicating the amount of time that it takes for one cycle of bioluminescence rhythms. The peak-to-trough intervals were measured for amplitude. Amplitude is determined by multiple factors such as cell number, circadian reporter expression, and cell communication. Since cell number and transfection were well controlled, amplitude may indicate the level of synchronization in this study. The specific time point when bioluminescence reached the highest amplitude was measured as phase. Furthermore, the software also estimated the number of days required for the rhythm amplitude to decrease to 36% of the initial value as damping rate, and the percentage the observed rhythms fit the expected sine wave as goodness of fit indicating stability and robustness of the rhythms ^49^.

### Wheel-running activity

C57BL/6J (males: n = 7; females: n = 7), A/J (males: n = 8; females: n = 8), and CAST/EiJ (males: n = 8; females: n = 6–8), mice (8–12 weeks old) were maintained on a 12:12 light–dark cycle (lights on at 7:00 am) for ~ 2 weeks and then were released into constant darkness for an additional 4–6 weeks to measure free-running activity rhythms. Chi-square periodogram analysis (ClockLab, ActiMetrics) was used to determine the period and amplitude of free-running rhythms.

### Statistical analysis

All statistical analyses were completed using Prism software (GraphPad). Analysis of variance (ANOVA) tests followed by Bonferroni’s multiple comparisons tests were performed to estimate strain and sex effects. Heritability estimates were calculated as the percentage of variability caused by strain differences from linear models using the isogenic strain as the independent categorical variable:

$${\mathrm{h}}^{2}=\frac{{\mathrm{MS}}_{\mathrm{strain}}}{{\mathrm{MS}}_{\mathrm{strain}}+\left({\mathrm{n}}_{\mathrm{mean}}-1\right)\times {\mathrm{MS}}_{\mathrm{resid}}}$$

where MS_strain_ indicates the mean square of the strain effect, n_mean_ indicates the mean number of samples within each strain, and MS_resid_ indicates the mean square of the residuals. For correlations between period and phase in DO mice and their eight founder strains, the period values were transformed using square root because the distributions were positively skewed.

## Supplementary Information

Supplementary Figures.

## References

[1] Takahashi JS (2017). Transcriptional architecture of the mammalian circadian clock. Nat. Rev. Genet..

[2] Zhang R, Lahens NF, Ballance HI, Hughes ME, Hogenesch JB (2014). A circadian gene expression atlas in mammals: Implications for biology and medicine. Proc. Natl. Acad. Sci. USA..

[3] Siepka SM, Takahashi JS (2005). Forward genetic screens to identify circadian rhythm mutants in mice. Methods Enzymol..

[4] Funato H (2020). Forward genetic approach for behavioral neuroscience using animal models. Proc. Jpn. Acad. Ser. B Phys. Biol. Sci..

[5] Logan RW (2013). High-precision genetic mapping of behavioral traits in the diversity outbred mouse population. Genes Brain Behav..

[6] Keenan BT (2020). High-throughput sleep phenotyping produces robust and heritable traits in diversity outbred mice and their founder strains. Sleep.

[7] Gottlieb, D. J., O'Connor, G. T. & Wilk, J. B. Genome-wide association of sleep and circadian phenotypes. *BMC Med. Genet.* **8 Suppl 1**, S9, 10.1186/1471-2350-8-S1-S9 (2007). 10.1186/1471-2350-8-S1-S9 PMC1995620 17903308

[8] Klei L (2005). Heritability of morningness-eveningness and self-report sleep measures in a family-based sample of 521 hutterites. Chronobiol. Int..

[9] Madrid-Valero JJ, Rubio-Aparicio M, Gregory AM, Sanchez-Meca J, Ordonana JR (2020). Twin studies of subjective sleep quality and sleep duration, and their behavioral correlates: Systematic review and meta-analysis of heritability estimates. Neurosci. Biobehav. Rev..

[10] Zhang EE (2009). A genome-wide RNAi screen for modifiers of the circadian clock in human cells. Cell.

[11] Maier B (2009). A large-scale functional RNAi screen reveals a role for CK2 in the mammalian circadian clock. Genes Dev.

[12] Hastings MH (2005). Circadian biology: Fibroblast clocks keep ticking. Curr. Biol..

[13] Brown SA (2005). The period length of fibroblast circadian gene expression varies widely among human individuals. PLoS Biol..

[14] Takahashi JS, Shimomura K, Kumar V (2008). Searching for genes underlying behavior: Lessons from circadian rhythms. Science.

[15] Chen Z, Yoo SH, Takahashi JS (2013). Small molecule modifiers of circadian clocks. Cell Mol. Life Sci..

[16] McCarthy MJ, Fernandes M, Kranzler HR, Covault JM, Welsh DK (2013). Circadian clock period inversely correlates with illness severity in cells from patients with alcohol use disorders. Alcohol Clin. Exp. Res..

[17] Yang S, Van Dongen HP, Wang K, Berrettini W, Bucan M (2009). Assessment of circadian function in fibroblasts of patients with bipolar disorder. Mol. Psychiatry.

[18] McCarthy MJ (2013). Genetic and clinical factors predict lithium's effects on PER2 gene expression rhythms in cells from bipolar disorder patients. Transl. Psychiatry.

[19] Yagita K, Okamura H (2000). Forskolin induces circadian gene expression of rPer1, rPer2 and dbp in mammalian rat-1 fibroblasts. FEBS Lett..

[20] Eastman CI, Suh C, Tomaka VA, Crowley SJ (2015). Circadian rhythm phase shifts and endogenous free-running circadian period differ between African-Americans and European-Americans. Sci. Rep..

[21] Wright KP, Gronfier C, Duffy JF, Czeisler CA (2005). Intrinsic period and light intensity determine the phase relationship between melatonin and sleep in humans. J. Biol. Rhythms.

[22] Jones CR (1999). Familial advanced sleep-phase syndrome: A short-period circadian rhythm variant in humans. Nat. Med..

[23] Micic G (2013). The endogenous circadian temperature period length (tau) in delayed sleep phase disorder compared to good sleepers. J. Sleep Res..

[24] Kadener S, Menet JS, Schoer R, Rosbash M (2008). Circadian transcription contributes to core period determination in Drosophila. PLoS Biol..

[25] Izumo M, Johnson CH, Yamazaki S (2003). Circadian gene expression in mammalian fibroblasts revealed by real-time luminescence reporting: Temperature compensation and damping. Proc. Natl. Acad. Sci. U S A.

[26] Farnell YF, Shende VR, Neuendorff N, Allen GC, Earnest DJ (2011). Immortalized cell lines for real-time analysis of circadian pacemaker and peripheral oscillator properties. Eur. J. Neurosci..

[27] Welsh DK, Yoo SH, Liu AC, Takahashi JS, Kay SA (2004). Bioluminescence imaging of individual fibroblasts reveals persistent, independently phased circadian rhythms of clock gene expression. Curr. Biol. (CB).

[28] Chen Z (2012). Identification of diverse modulators of central and peripheral circadian clocks by high-throughput chemical screening. Proc. Natl. Acad. Sci. USA..

[29] Keane TM (2011). Mouse genomic variation and its effect on phenotypes and gene regulation. Nature.

[30] Konopka RJ, Benzer S (1971). Clock mutants of *Drosophila melanogaster*. Proc. Natl. Acad. Sci. U S A.

[31] Zhang L, Ptacek LJ, Fu YH (2013). Diversity of human clock genotypes and consequences. Prog. Mol. Biol. Transl. Sci..

[32] Duong ATH (2019). The clock mechanism influences neurobiology and adaptations to heart failure in clock(19/19) mice with implications for circadian medicine. Sci. Rep..

[33] Kalmbach DA (2017). Genetic basis of chronotype in humans: Insights from three landmark GWAS. Sleep.

[34] Jiang P, Striz M, Wisor JP, O'Hara BF (2011). Behavioral and genetic dissection of a mouse model for advanced sleep phase syndrome. Sleep.

[35] Hofstetter JR, Svihla-Jones DA, Mayeda AR (2007). A QTL on mouse chromosome 12 for the genetic variance in free-running circadian period between inbred strains of mice. J. Circadian Rhythms.

[36] Yang HS, Vitaterna MH, Laposky AD, Shimomura K, Turek FW (2009). Genetic analysis of daily physical activity using a mouse chromosome substitution strain. Physiol. Genomics.

[37] Wax TM (1977). Effects of age, strain, and illumination intensity on activity and self-selection of light-dark schedules in mice. J. Comp. Physiol. Psychol..

[38] Liu AC (2007). Intercellular coupling confers robustness against mutations in the SCN circadian clock network. Cell.

[39] Landgraf D, Wang LL, Diemer T, Welsh DK (2016). NPAS2 compensates for loss of CLOCK in peripheral circadian oscillators. PLoS Genet..

[40] Schoenrock SA (2020). Characterization of genetically complex collaborative cross mouse strains that model divergent locomotor activating and reinforcing properties of cocaine. Psychopharmacology.

[41] Noguchi T, Wang LL, Welsh DK (2013). Fibroblast PER2 circadian rhythmicity depends on cell density. J. Biol. Rhythms.

[42] Koritala BSC, Lee K (2017). Natural variation of the circadian clock in neurospora. Adv. Genet..

[43] 43von Schantz, M. Natural variation in human clocks. *Adv Genet* **99**, 73–96, 10.1016/bs.adgen.2017.09.003 (2017). 10.1016/bs.adgen.2017.09.003 29050555

[44] Sawyer LA (1997). Natural variation in a Drosophila clock gene and temperature compensation. Science.

[45] Marcheva B (2010). Disruption of the clock components CLOCK and BMAL1 leads to hypoinsulinaemia and diabetes. Nature.

[46] McClung CA (2005). Regulation of dopaminergic transmission and cocaine reward by the Clock gene. Proc. Natl. Acad. Sci. U S A.

[47] Leng Y, Musiek ES, Hu K, Cappuccio FP, Yaffe K (2019). Association between circadian rhythms and neurodegenerative diseases. Lancet Neurol..

[48] Menger, G. J. *et al.* Circadian profiling of the transcriptome in NIH/3T3 fibroblasts: comparison with rhythmic gene expression in SCN2.2 cells and the rat SCN. *Physiol. Genomics* **29**, 280–289, 10.1152/physiolgenomics.00199.2006 (2007). 10.1152/physiolgenomics.00199.2006 17284666

[49] Izumo M, Sato TR, Straume M, Johnson CH (2006). Quantitative analyses of circadian gene expression in mammalian cell cultures. PLoS Comput. Biol..

