# Supplementary material for: High-throughput measurement of fibroblast rhythms reveals genetic heritability of circadian phenotypes in diversity outbred mice and their founder strains
Source: Sci Rep. 2021 Jan 28;11:2573. doi: 10.1038/s41598-021-82069-8 (PMC7843998; doi:10.1038/s41598-021-82069-8)
Supplement: Supplementary file 1 — Supplementary Figures. [file 41598_2021_82069_MOESM1_ESM.docx]

**Supplementary Figure 1.** Representative bioluminescence recordings of ensemble *Bmal1-dLuc* rhythms in two individual fibroblast cultures derived from eight founder strains by raw data.

**Supplementary Figure 2.** Correlations between period and phase of fibroblast rhythms in DO and their founder strains.

**Supplementary Fig 1**

**
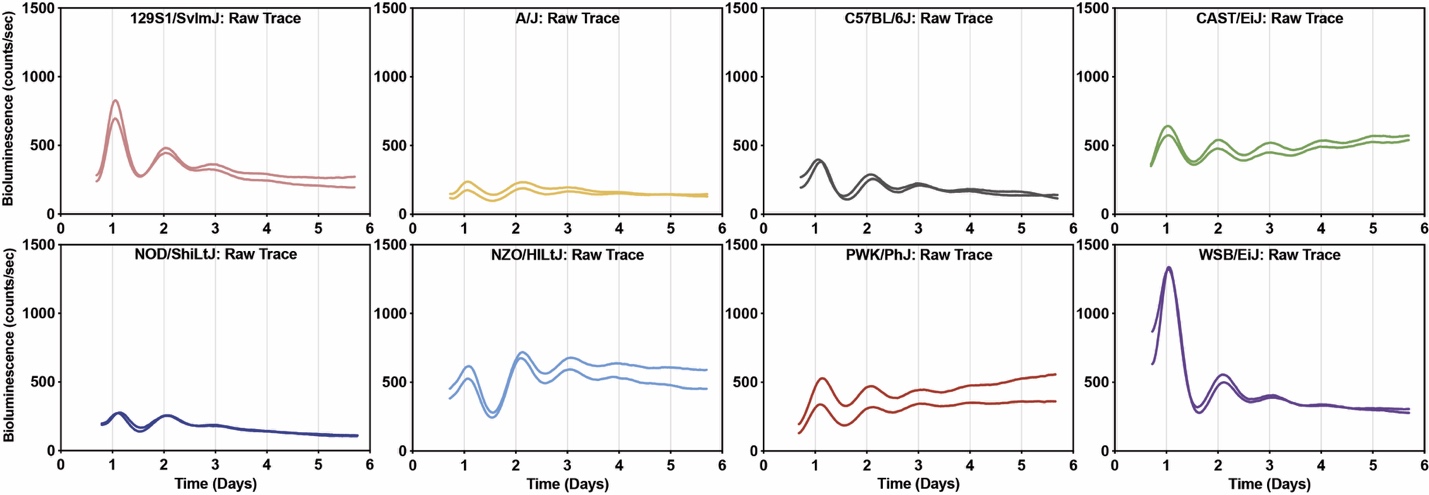
**

**Supplementary Fig 2**

**
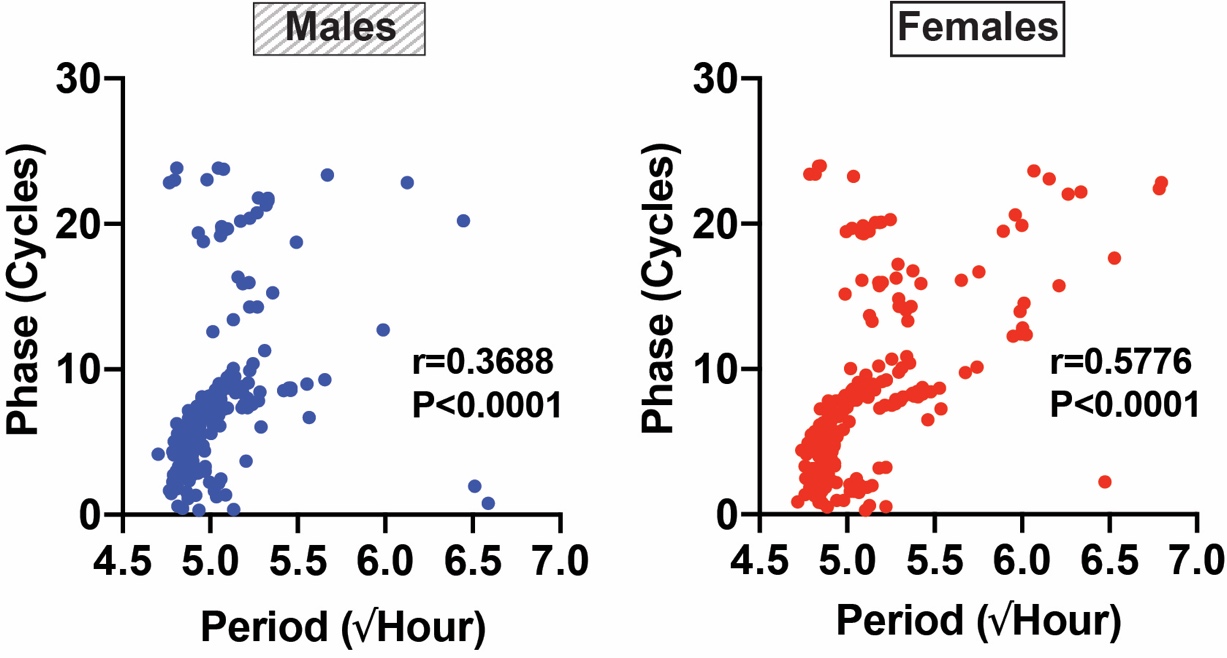
**
